# The Binding of HSPA8 and Mitochondrial ALDH2 Mediates Oxygen-Glucose Deprivation-Induced Fibroblast Senescence

**DOI:** 10.3390/antiox13010042

**Published:** 2023-12-25

**Authors:** Wenting Hui, Tongtong Song, Ling Yu, Xia Chen

**Affiliations:** 1 Department of Pharmacology, College of Basic Medical Sciences, Jilin University, Changchun 130012, China; huiwt21@mails.jlu.edu.cn; 2 Department of Anatomy, College of Basic Medical Sciences, Jilin University, Changchun 130012, China; songtongtong@jlu.edu.cn; 3 Department of Pharmacy, The Second Hospital of Jilin University, Changchun 130022, China; yulingyxb@jlu.edu.cn

**Keywords:** cellular senescence, oxygen-glucose deprivation, ALDH2, HSPA8

## Abstract

Cellular senescence refers to the permanent and irreversible cessation of the cell cycle. Recently, it has gained significant interest as a promising target for preventing cardiovascular diseases. Aldehyde dehydrogenase 2 (ALDH2) is a mitochondrial enzyme that has been closely linked with an increased risk of cardiovascular diseases. In this study, bioinformatics analysis revealed that the signaling pathway for fibroblast senescence is significantly activated in mice after myocardial infarction (MI), and that ALDH2 might be a crucial molecule responsible for inducing this change. Therefore, we created an NIH3T3 fibroblast cell line oxygen-glucose deprivation (OGD) model to replicate the conditions of MI in vitro. We further revealed that decreased ALDH2 enzyme activity is a critical factor that affects fibroblast senescence after OGD, and the activation of ALDH2 can improve the mitochondrial damage caused by OGD. We identified Heat Shock 70-kDa Protein 8 (HSPA8) as an interacting protein of ALDH2 through co-immunoprecipitation (Co-IP) and mass spectrometry (MS) detection. Subsequently, our studies showed that HSPA8 translocates to the mitochondria after OGD, potentially binding to ALDH2 and inhibiting its enzyme activity. By transfecting siRNA to inhibit HSPA8 expression in cells, it was found that ALDH2 enzyme activity can be significantly increased, and the senescence characteristics induced by OGD in NIH3T3 cells can be improved. In conclusion, the data from this study suggest that HSPA8, in conjunction with ALDH2, could regulate fibroblast senescence after oxygen-glucose deprivation, providing a new direction and foundation for effectively intervening in fibroblast senescence after myocardial infarction.

## 1. Introduction

Myocardial infarction (MI) is a leading cause of death among non-communicable diseases worldwide [1]. Despite advancements in medical and surgical treatments, the clinical outcomes for MI patients remain poor. This situation may be attributed to the complex mechanisms of myocardial injury [2]. Numerous fibroblasts are present in the myocardium of adult mammals [3]. Following a myocardial infarction, cardiac fibroblasts experience changes in their phenotype that aid in controlling inflammation, repairing damage, and promoting an angiogenesis response [4,5]. Understanding the impact of MI on the fate of fibroblasts can contribute to developing innovative therapies.

Recent research has shown that the senescence of various cell types in the heart is linked to different cardiovascular diseases (CVDs). Cellular senescence is characterized by an irreversible arrest of the cell cycle, which can be triggered by negative stressors such as aging, DNA damage, or heightened levels of reactive oxygen species (ROS) [6]. The p53/p21 and p16Ink4a/retinoblastoma signaling pathways are activated in most senescent cells, and senescence-associated β-galactosidase (SA-β-gal) is widely used to identify senescent cells [7]. Additionally, senescent cells can exhibit senescence-associated secretory phenotypes (SASPs), where soluble signaling factors, proteases, and insoluble protein/extracellular matrix (ECM) components are secreted into the extracellular space and impact the surrounding cells through paracrine effects [8]. While the transient presence of senescent cells in the heart may be beneficial in response to temporary stress, the long-term accumulation of senescent cells can impair cardiac function [6]. The pharmacological clearance of senescent cells has been shown to enhance myocardial repair after acute myocardial infarction in aging mice and significantly improve mouse survival rates [9,10]. Therefore, therapeutic interventions that focus on senescent cells have the potential to improve the disease prognosis and alleviate cardiac dysfunction [11].

Studies have found that the levels of cell senescence biomarkers in the hearts of mice are notably elevated after a heart attack [12]. During cardiac remodeling in a mouse model of transverse aortic contraction (TAC), it was observed through double staining of p21 and fibroblast markers (PDGFα, vimentin, and α-SMA) that senescent cells included both fibroblasts and myofibroblasts [13]. The premature senescence of cardiac fibroblasts can hinder collagen expression and restrict reparative fibrosis during the initial stages of wound healing [6]. Senescent fibroblasts also release SASPs factor, which can negatively impact ECM remodeling and increase the likelihood of heart rupture [14]. Nonetheless, the exact regulatory mechanism of fibroblast senescence following MI remains unclear.

ALDH2 is an aldehyde oxidase located in mitochondria and is primarily present in organs such as the liver, heart, lung, and kidney, which are rich in mitochondria [15]. Several studies have shown that the function of ALDH2 may positively affect cardiovascular health [16]. The pharmacological enhancement of ALDH2 activity after myocardial infarction could improve ischemic heart injury [17,18]. It has been discovered in recent years that ALDH2 has the ability to prevent myocardial senescence that is not related to age by having an anti-cellular senescence effect [19]. When the activity of ALDH2 is reduced, it can lower the oxygen reserve capacity of endothelial cell mitochondria, leading to premature senescence of these cells [20]. On the other hand, activating ALDH2 can protect against endothelial senescence and atherosclerosis [21]. However, the molecular mechanism behind fibroblast senescence induced by ALDH2 has yet to be comprehensively understood.

Mitochondrial damage plays a crucial role in the development and pathogenesis of cardiovascular diseases. Mitochondrial dynamics serves as the primary mechanism that controls the cellular mitochondrial health under both physiological and pathophysiological environments [22]. Changes in mitochondrial dynamics are also recognized as an important factor regulating cellular senescence [23,24]. Recent research has focused on the role of ALDH2 in improving mitochondrial dynamic abnormalities. According to Tan et al., the ALDH2 pathway regulates mitochondrial fusion and fission in diabetic cardiomyopathy to alleviate ischemia and reperfusion injury [25]. Similarly, Zhao et al. found that ALDH2 attenuates the development of hypoxia-induced pulmonary hypertension by regulating mitochondrial fission and smooth muscle cell proliferation [26]. However, it is not clear whether ALDH2 plays a role in mitochondrial dynamics during cellular senescence.

HSPA8 belongs to the heat shock protein 70 family and has a variety of cellular functions, participating in various cellular events such as apoptosis, ubiquitination, autophagy, and signal transduction [27,28,29]. As a member of the heat shock family, HSPA8 can help maintain the standard structure and function of other proteins when cells are subjected to inappropriate environments, and its abnormal expression may lead to various diseases [30]. However, the regulation of HSPA8 on cell senescence needs more research. 

In order to further understand the endogenous mechanism of fibroblast senescence after MI, we developed a cell oxygen-glucose deprivation (OGD) model, which is similar to the MI environment [31]. We have for the first time shown that the interaction between HSPA8 and ALDH2 after OGD may play an important role in regulating fibroblast senescence.

## 2. Methods

### 2.1. Bioinformatics Analysis

Two raw datasets (GSE111059 [32] and GSE28416 [33]) containing gene expression data for cardiac fibroblasts post-MI and mouse embryo fibroblast were downloaded from the Gene Expression Omnibus (GEO). Differential expression genes (DEGs) were extracted and analyzed using the GEO2R online analysis tool (https://www.ncbi.nlm.nih.gov/geo/geo2r/, accessed on 20 October 2022 and 28 November 2022), an R-based web application included in the GEO database. The DEGs obtained from the two datasets were visualized using the R packages “complexheatmap” and “ggplot2” to generate heat maps and volcano maps, respectively. The mass spectrometry proteomics data were obtained from the ProteomeXchange Consortium (http://www.proteomexchange.org/; accessed on 22 October 2022) under the dataset identifier PXD021469 [34]. Single-cell sequencing data of mouse myocardial tissue were derived from Tabula Muris and visualized using online tools available at https://tabula-muris.ds.czbiohub.org/ (accessed on 29 November 2022) [35]. The differences between gene lists were obtained from different differential analyses using JVENN [36]. To further analyze the gene sets, gene set enrichment analysis (GSEA) and functional enrichment analysis were conducted using the R package “clusterProfiler”. Results were visualized using an online platform for data analysis and visualization, https://www.bioinformatics.com.cn (accessed on 28 October 2022 and 29 November 2022).

### 2.2. Chemicals and Reagents

ALDH2-seletived agonist Alda-1 (99.99% purity) and inhibitor CVT10216 (99.0% purity) were obtained from MedChemExpress (Monmouth Junction, NJ, USA). Anti-HIF-1α (AF1009), p53 (AF0879) and p21 (AF6290) antibodies were purchased from Affinity Bioscience (Cincinnati, OH, USA). Anti-CDKN2α/p16INK4a antibody (A0262) was purchased from ABclonal (Wuhan, China). Anti-ALDH2 (15310-1-AP), Drp1 (12957-1-AP), Fis1 (10956-1-AP), Mfn1 (13798-1-AP), Mfn2 (12186-1-AP), and HSPA8 (10654-1-AP) antibodies were purchased from Proteintech (Chicago, IL, USA). GAPDH (HC301-01) antibody was purchased from TransGen Biotech (Beijing, China). Anti-BAX (bs-28034R), Goat anti-rabbit-IgG (bs-0295G-HRP), and Goat-anti-mouse-IgG antibodies (bs-0296G-HRP) were purchased from Bioss (Beijing, China). Alexa Fluor 488-labeled Goat Anti-Rabbit IgG and Normal Rabbit IgG antibodies were purchased from Beyotime (Shanghai, China). 

### 2.3. Cell Culture 

Mouse fibroblast cell line NIH3T3 was obtained from Shanghai Zhong Qiao Xin Zhou Biotechnology Co., Ltd. (Shanghai, China). They were grown in Dulbecco’s Modified Eagle Medium supplemented with 10% heat-inactivated fetal bovine serum. The cells were cultivated at 37 °C in a humidified atmosphere containing 5% CO_2_ and 95% air.

### 2.4. Oxygen and Glucose Deprivation Cell Model

Oxygen and glucose deprivation were performed in NIH3T3 cell lines as previously reported [37]. Briefly, cells were washed once with PBS and the medium was then replaced with glucose-free DMEM. Then, cells were transferred to an hypoxic chamber containing a mixture gas composed of 94% N_2_, 1% O_2_ and 5% CO_2_, while the control group was maintained in an aerobic environment (95% air and 5% CO_2_).

### 2.5. Quantitative Reverse Transcription PCR (RT-qPCR)

Total RNA was isolated from rat hearts by using TransZol Up (TransGen Biotech, Shanghai, China, ET111-01-V2). Template RNA were reverse transcribed using TransScript^®^ First-Strand cDNA Synthesis SuperMix Kit (TransGen Biotech, Shanghai, China, AT301-02). The mRNA levels were measured using SYBR green (Roche, Basel, Switzerland, 491391400) real-time PCR. Primer sequences were as shown in Table 1.

### 2.6. Western Blot Analysis

Briefly speaking, tissue protein extraction and Western blotting were carried out as described [38]. Protein mixtures were normalized to 20 µg and loaded into each well and separated using 10–15% sodium dodecyl sulfate (SDS) polyacrylamide gel electrophoresis. Following a 120 min run, the proteins were then electrophoretically transferred onto PVDF membranes (Millipore, New Jersey, NJ, USA, IPVH00010). Then, membranes were immersed in the blocking buffer containing 5% skimmed milk (BD, Franklin Lakes, NJ, USA, 232100) for 2 h prior to the incubation with primary antibodies. Horseradish peroxidase-conjugated secondary antibodies were correspondingly used to incubate the membranes for 1 h. Bands were visualized under the Tanon chemiluminescence system. Results were processed using ImageJ2 software.

### 2.7. Senescence-Associated β-Galactosidase (SA-β-gal) Staining and Activity Assay

SA-β-gal positive cells were identified using an β-gal stain kit (Solarbio, Beijing, China, 232100) according to the manufacturer’s instructions. Briefly, cells were washed with phosphate-buffered saline (PBS) and fixed with 1X fixative for 15 min at room temperature. The β-galactosidase staining solution was added to the plates and incubated overnight in a dry incubator at 37 °C without CO_2_. The next day, the β-Gal positive cells were observed under a light microscope (OLYMPUS). In addition, SA-β-gal activity assay was performed using an β-gal activity assay kit (Solarbio, Beijing, China, BC2585). β-gal decomposes p-nitrobenzene-β-D-galactoside pyranopyranoside to p-nitrophenol, which has a maximum absorption peak at 400 nm. β-gal activity was calculated by measuring the rate of increase of absorption value.

### 2.8. Cell Proliferation Assay

The manufacturer’s instructions were followed to identify ki67-positive cells using a Ki67 Cell Proliferation Assay Kit (Beyotime, Shanghai, China, C2301S). The cells were washed with PBS and fixed with fixative for 15 min at room temperature. Next, the cells were cleaned with washing solution and the immunostaining blocking solution was added and left to incubate at room temperature for 10–20 min. The Ki67 rabbit monoclonal antibody was then added and incubated at 4 °C overnight. After washing the cells, anti-rabbit Cy3 was added and incubated at room temperature for 1 h. Finally, the nuclear staining solution (DAPI) was added and left to stain at room temperature for about 5 min. After the cover glass was sealed, it was observed using a fluorescence microscope.

### 2.9. Measurement of Glycolysis Rate

The glycolysis rate was measured using Glycolysis Assay Kit (Abcam, Cambridge, UK, ab197244). The appropriate number of cells was carefully planted in a 96-well plate. Post-treatment, the supernatant was removed, and the cells were thoroughly washed twice with 100 μL respiratory buffer. Subsequently, 150 μL respiratory buffer was delicately added to each well, followed by the addition of 10 μL glycolysis detection reagent. The glycolysis rate of the cell was detected using a BioTek Gen5 Microplate Reader (Winooski, VT, USA).

### 2.10. ALDH2 Enzyme Activity Assay

The activity of ALDH2 was measured using a Mitochondrial Aldehyde Dehydrogenase Activity Assay Kit (Solarbio, Beijing, China, BC0755). ALDH2 catalyzes the conversion of acetaldehyde and NAD+ to acetic acid and NADH, so the activity of ALDH2 can be calculated by the change in the absorption value of NADH at 340 nm.

### 2.11. Mitochondrial Membrane Potential Assay

The mitochondrial membrane potential (ΔΨ m) was detected using JC-1 (Solarbio, Beijing, China, M8650). Briefly, cells were washed twice in PBS, stained with JC-1 for 20 min at 37 °C, washed twice with staining buffer, and imaged using an OLYMPUS IX71 inverted fluorescence microscope (Tokyo, Japan).

### 2.12. Measurement of ROS and ATP

ROS content was measured using a ROS detection kit (Solarbio, Beijing, China, CA1410) according to the manufacturer’s instructions. Cells were washed twice in PBS, incubated with DCFH-DA for 20 min at 37 °C. The ROS content of the cell was detected using a BioTek Gen5 Microplate Reader (VT, USA).

ATP levels were measured using an ATP assay kit (Solarbio, Beijing, China, BC0305) according to the manufacturer’s instructions. First, we collected cell lysates with lysis buffer, centrifuged at 10,000× *g* for 5 min at 4 °C, and then transferred the supernatant and added chloroform and shook it thoroughly. After centrifugation, the supernatant was added to the working fluid for detecting. Luminescence was detected using a multifunctional microplate reader (Thermo Fisher Scientific, Waltham, MA, USA). 

### 2.13. Co-Immunoprecipitation (Co-IP) Assay

Co-IP assay was accomplished by using a Co-IP kit (Absin, Shanghai, China, abs955) under the manufacturer’s illustrations. The cells were washed once with PBS and lysed in lysis buffer on ice for 30 min. The soluble cell lysate factions were collected and centrifugated at 4 °C under 14,000× *g* for 10 min. After that, part of cell lysate was selected as the positive control. The other remaining protein fractions were soaked with rabbit anti-ALDH2 antibody and rabbit IgG antibody overnight at 4 °C, followed by being mixed gently with protein A + G agarose beads at 4 °C for 3 h. Wash buffer was used to rinse the beads five times. At last, HSPA8 antibody were tested using Western blot.

### 2.14. Mass Spectrometry

NIH3T3 cells were stimulated with OGD for 4 h and baited with anti-ALDH2 antibody. We collected protein for Co-IP as described above. Successful immunoprecipitation of ALDH2 was verified using Coomassie blue staining and Western blot. The immunoprecipitant protein was then subjected to liquid chromatography with tandem mass spectrometry for proteomics analysis.

### 2.15. Immunofluorescence Staining

NIH3T3 cells were cultured in 12-well plates. The cells were treated with OGD for 4 h and stained with MitoTracker™ Red CMXRos (Invitrogen, Waltham, MA, USA, M7512) for 15 min at 25 °C. Then, we washed the cells three times with PBS. The cells were fixed with Immunol Staining Fix Solution (Beyotime, Shanghai, China, P0098) for 30 min and bathed with PBS. Then, 0.2% Triton X-100 was added to the medium at room temperature for 20 min. The cells were blocked with 5% bovine serum albumin (BSA, Solarbio, Beijing, China, A8020) for 30 min and then incubated with primary antibodies at 4 °C overnight. After the cells were washed with PBS, the excess liquid was sucked up using absorbent paper and the cells were incubated with Alexa Fluor 488-labeled Goat Anti-Rabbit IgG secondary antibody at 37 °C for 1 h. The nuclei were stained with DAPI (Beyotime, Shanghai, China C1005) for 5 min. Photographs were taken under fluorescence microscopy (ZEISS, Oberkochen, Germany).

### 2.16. Extraction of Cytoplasmic and Mitochondrial Proteins

After the treatment described above, cytosolic and mitochondrial proteins were detached by using the Mitochondrial and Cytoplasmic Protein Extraction Kit (Nanjing Jiancheng, Nanjing, China, G008-1-1). After protein quantification, the variation of target proteins expression was detected via Western blotting.

### 2.17. Transfection of siRNA

HSPA8-specific siRNA molecules were chemically synthesized by Guangzhou RiboBio Co., Ltd. (Guangzhou, China). The non-specific siRNA (scramble) consisted of a non-target used as a control. Transfection was performed by utilizing Lipofectamine 3000 reagent as per the manufacturer’s protocol. The transfection efficiency was detected using RT-qPCR.

### 2.18. Disuccinimidyl Suberate (DSS) Crosslinking

Crosslinking was performed according to instructions of disuccinimidyl suberate (DSS) crosslinkers (Thermo Scientific, Waltham, MA, USA) [39]. Cells were collected and washed two times with ice-cold PBS. We added the DSS solution to a final concentration of 500 μΜ, and incubated the reaction mixture for 30 min at room temperature. We added the Quench Solution to a final concentration of 10 mM Tris and incubated the quenching reaction for 15 min at room temperature. Lysates were analyzed using Western blot.

### 2.19. Statistical Analysis

All of the data are expressed as the mean ± standard deviation (SD). The differences between data sets were assessed using Student’s *t*-test and analysis of variance (ANOVA) with Holm–Sidak’s multiple comparison test. The figures were plotted using GraphPad Prism 9.0. Levels of statistical significance are indicated in the figures using asterisks and other symbols (* *p* < 0.05, ** *p* < 0.01, *** *p* < 0.001). Each experiment was conducted at least in triplicate.

## 3. Results

### 3.1. Identification and Functional Enrichment Analysis of DEGs in Mice CFs after MI 

**Figure 1:**
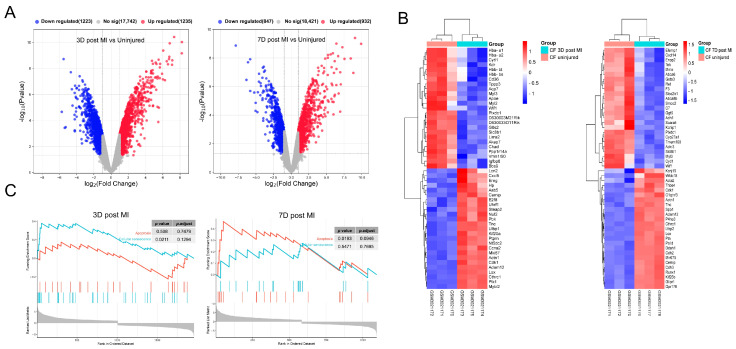
Identification and Functional Enrichment Analysis of differentially expressed genes (DEGs) in mice cardiac fibroblasts (CFs) after myocardial infarction (MI). (**A**) Respective volcano plots of sifted out DEGs for datasets in accordance with public database GSE111059. Red and Blue in the plot represent up- and down-regulated genes, respectively. Blank plots represent the remaining genes with no significant difference. (**B**) Respective heat map of sifted out DEGs for datasets in accordance with public database GSE111059. (**C**) Gene Set Enrichment Analysis (GSEA) of DEGs.

The gene expression profile GSE111059 in the NCBI-GEO public data set was analyzed to identify the DEGs in myocardial fibroblasts of myocardial infarction mice and control mice. After data processing, a total of 2458 DEGs were obtained 3 days after MI. The MI group displayed 1235 up-regulated genes and 1223 down-regulated genes compared to the uninjured group. A total of 1779 DEGs were obtained after 7 days of MI. In the MI fibroblast group, there were 932 up-regulated genes and 847 down-regulated genes compared to the uninjured group. Volcanic maps and heat maps of differential genes are shown in Figure 1A,B. A GSEA of the DEGs was performed using the clusterprofiler R package. It is evident from Figure 1C that the cell fate exhibited variation at different time points during MI. At 3 days after MI, the DEGs showed significant enrichment in the cellular senescence pathway. At 7 days after MI, the DEGs were significantly enriched in the apoptotic pathway. These findings suggest that fibroblast senescence is induced during the early stages of myocardial infarction.

### 3.2. OGD Treatment-Induced Fibroblast Senescence

**Figure 2:**
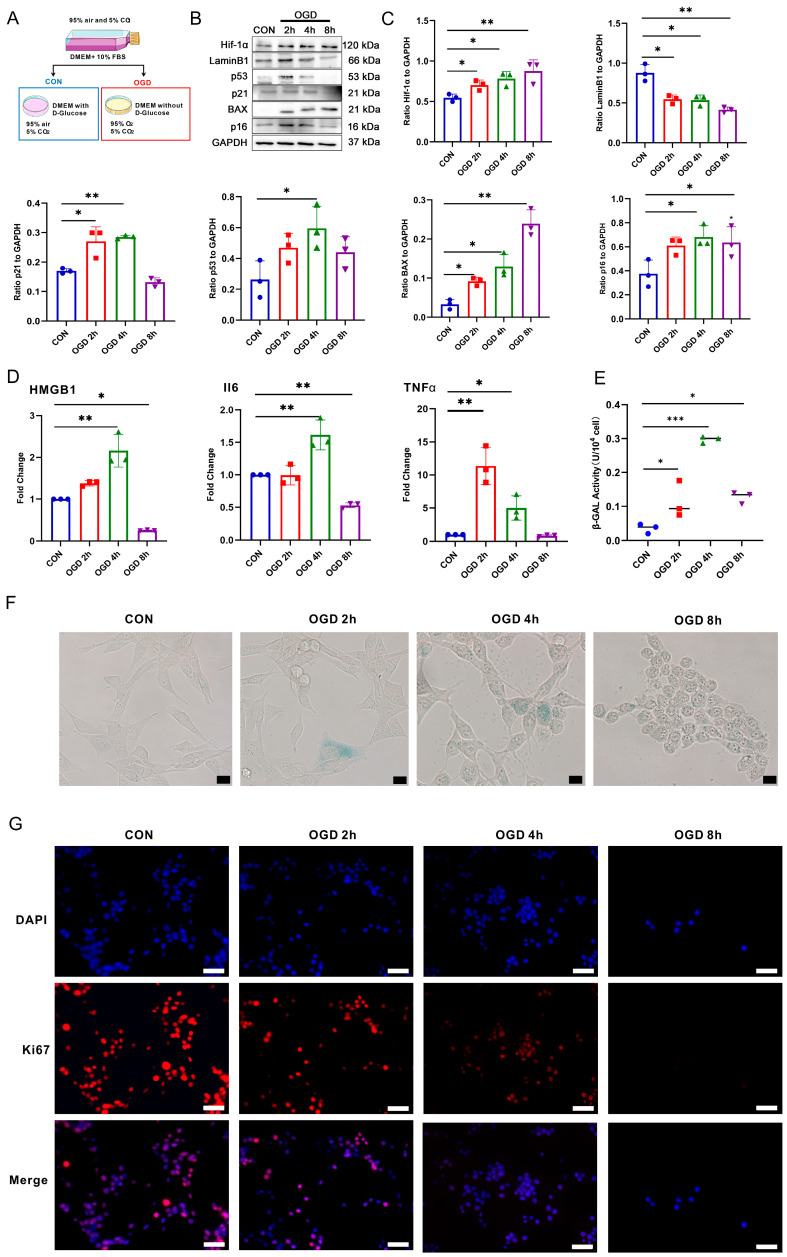
Effects of different duration of OGD on the fate of fibroblasts. (**A**) Cell grouping and treatment diagram. (**B**) Western blot analysis of Hif-1α, Lamin B1, p53, p21, p16, and BAX in NIH3T3 cells. (**C**) Quantification of (**B**). * *p* < 0.05, ** *p* < 0.01 compared with the CON group. (**D**) RT-qPCR analysis of HMGB1, IL6, and TNFα mRNA in NIH3T3 cells. * *p* < 0.05, ** *p* < 0.01 compared with the CON group. (**E**) β-galactosidase (β-GAL) activity analysis in NIH3T3 cells. * *p* < 0.05, *** *p* < 0.001 compared with the control group. (**F**) β-GAL staining of NIH3T3 cells. Scale bar = 50 μm. (**G**) Cell proliferation was detected using Ki67 staining. The red fluorescence in the image indicates the Ki67 positive cells, while the blue fluorescence indicates the nucleus. Scale bar = 50 μm.

To investigate the potential correlation between a deficiency in oxygen and glucose and the occurrence of fibroblast senescence, we conducted an in vitro experiment on NIH3T3 fibroblasts. As shown in Figure 2A, we subjected NIH3T3 fibroblasts to 37 °C, 1% O_2_, 5% CO_2_ culture conditions, and glucose-free DMEM medium. Based on the Western blot results in Figure 2B,C, it was noticeable that the cells treated with OGD had a higher expression of hypoxia-inducing factor Hif-1α than the control group. This result suggests that the hypoxia response was effectively triggered in the cells. The protein expressions of cell senescence markers p16, p21, and p53 were significantly increased 2 h after OGD and further increased 4 h after OGD. However, they were significantly decreased 8 h after OGD. The apoptosis marker BAX protein was significantly up-regulated after OGD for 8 h, indicating that the cells were more prone to apoptosis after OGD for 8 h. After 2 h of OGD, the expression of cell proliferating protein LaminB1 was significantly reduced, indicating that OGD resulted in a slowdown in cell proliferation. According to the results of RT-qPCR (as seen in Figure 2D), NIH3T3 cells subjected to OGD for 4 h displayed a notable increase in the expression of SASPs HMGB1, IL6, and TNFα mRNA when compared to the CON group. The results of detecting the intracellular β-GAL activity in NIH3T3 (Figure 2E) showed that β-GAL activity increased significantly after 4 h of OGD. The results of β-GAL staining showed (Figure 2F) that the number of β-GAL positive cells increased significantly after OGD for 4 h compared with the control group. Moreover, immunofluorescence staining was used to detect the expression of Ki67, a marker of cell proliferation [40]. The results (Figure 2G) showed that the quantity of Ki67 positive cells was significantly reduced in the OGD 2 h and 4 h groups than the control group. In addition, Ki67 expression was almost undetectable in the OGD 8 h group. These results suggest that OGD treatment inhibits fibroblast proliferation. Specifically, the incidence of cell senescence was more noticeable after 4 h of OGD, while cell apoptosis was more pronounced after 8 h of OGD.

### 3.3. Bioinformatics Analysis Revealed That ALDH2 Is a Potential Regulator of Fibroblast Senescence after OGD

**Figure 3:**
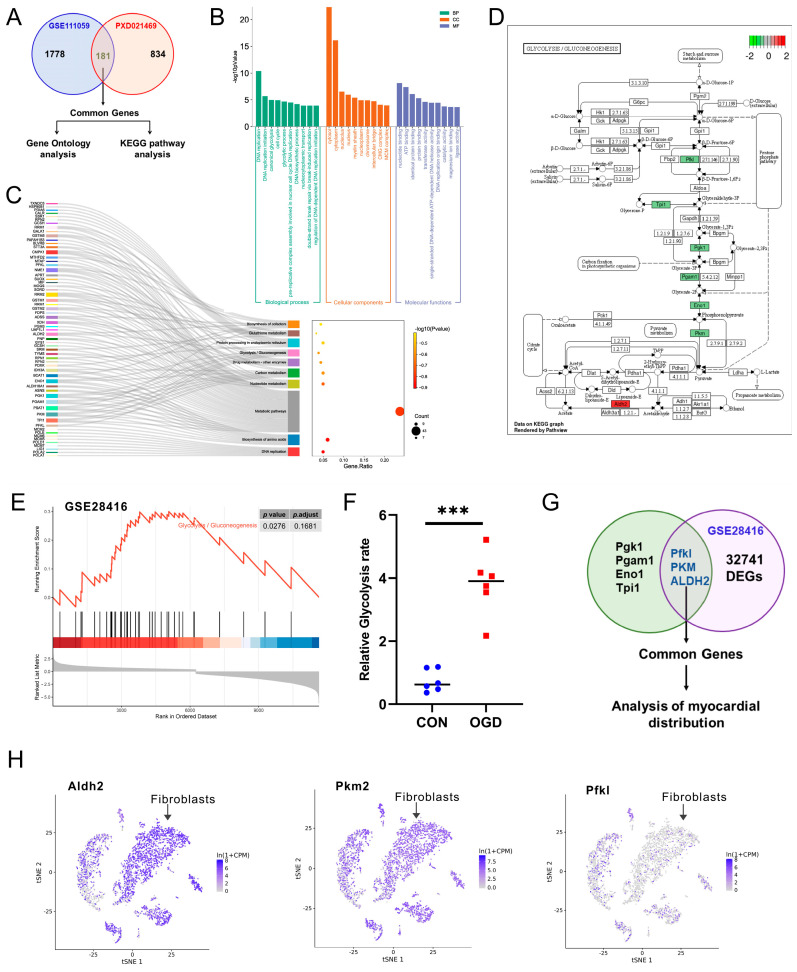
Bioinformatics analysis of DEGs. (**A**) The Venn diagrams of the overlapping homologous DEGs among the two datasets. (**B**) Histogram showing the top 10 GO events associated with DEGs of biological process (BP) module, molecular functions (MFs) module, and cellular components (CCs) module. (**C**) Sankey and dot plot showing the top 10 KEGG pathways associated with DEGs. (**D**) Gene expression in the glycolysis pathway. (**E**) GSEA analysis of DEGs in GSE28416 dataset. (**F**) Detection of glycolysis rate in NIH3T3 cells. *** *p* < 0.001 compared with the CON group. (**G**) The Venn diagrams of the overlapping homologous DEGs among the two kinds of analysis. (**H**) Expression and distribution of ALDH2, PKM, and PFKL genes in different cells of mouse myocardial tissue based on Tabula Muris database.

To determine the regulatory factors that may cause fibroblast senescence after OGD, we conducted a comprehensive analysis of myocardial fibroblast mRNA expression 3 days post-MI across the GSE111059 datasets and the NIH3T3 cells’ protein expression 4 h after OGD. The proteomic data were obtained from PXD021469 datasets in the ProteomeXchange Consortium [34]. We discovered that the mRNAs and proteins of 181 genes displayed significant differences following MI and OGD, as shown in Figure 3A. We performed a Metascape enrichment analysis of the 181 overlapping genes for GO and KEGG analysis. The *p*-value was used to determine the results of the correlation test, with the top ten of each category sorted in ascending order according to the LogP value, as shown in Figure 3B,C. We noticed that the top rank belonged to the DNA replication (GO: 0006260, KEGG: mmu03030) and glycolysis (GO: 0006096, KEGG: mmu00010) in Go and KEGG enrichment analysis. Seven DEGs (Pgk1, Pgam1, Eno1, Tpi1, Pfkl, PKM, ALDH2) related to the glycolysis pathway were identified within the group, as shown in Figure 3D. Then, we analyzed the gene expression profiles of mouse senescent fibroblasts induced through DNA damage from dataset GSE28416. The GSEA analysis of the DEGs in this dataset showed that the glycolysis pathway was activated in senescent fibroblasts (Figure 3E). As shown in Figure 3F, we detected the NIH3T3 cells’ glycolysis rate after 4 h of OGD treatment, and, compared with the CON group, the glycolysis rate increased significantly. In addition, we found that only ALDH2, PFKL, and PKM were differentially expressed in senescent fibroblasts among glycolysis-related genes (Figure 3G). Moreover, our analysis of single-cell sequencing data of mouse myocardial tissue revealed that ALDH2 was highly expressed in cardiac fibroblasts, as shown in Figure 3H and Figure S1. Hence, changes in ALDH2 expression may play a crucial role in MI- and OGD-induced fibroblast senescence.

### 3.4. Activation of ALDH2 Can Inhibit Fibroblast Senescence after OGD

**Figure 4:**
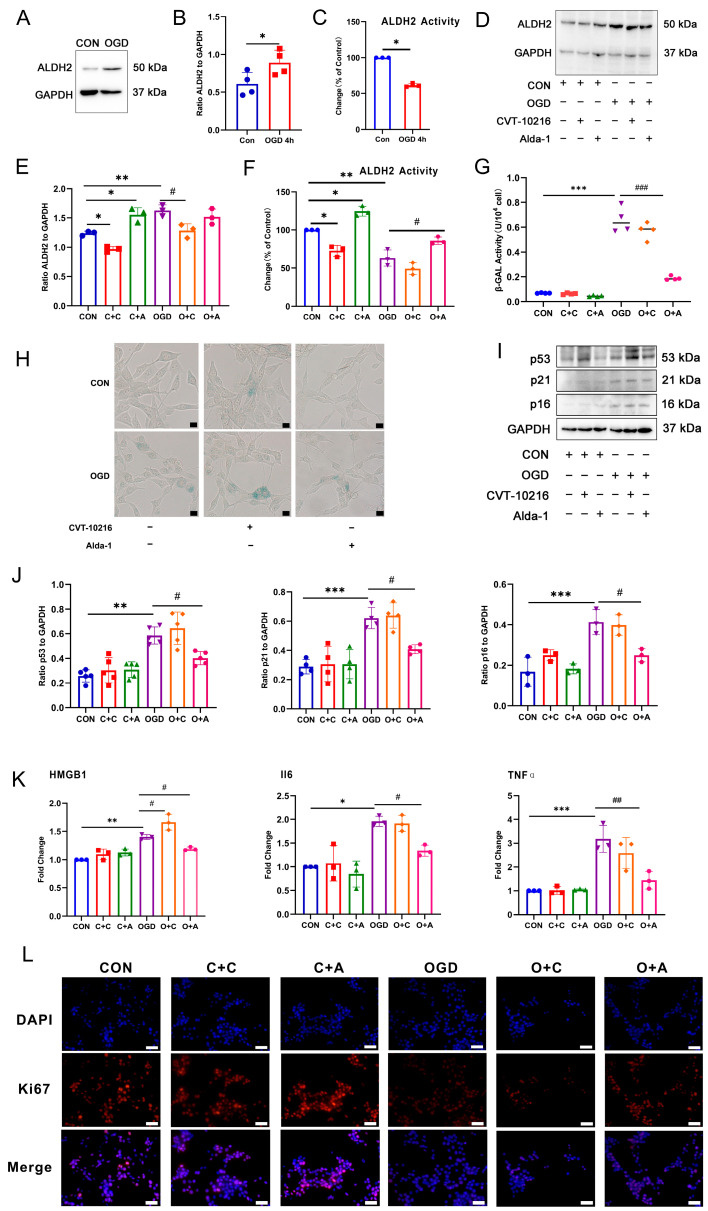
The effect of ALDH2 activity on fibroblast senescence after OGD. (**A**) Western blot analysis of ALDH2 in NIH3T3 cells. (**B**) Quantification of (**A**). * *p* < 0.05 compared with the CON group. (**C**) Changes in ALDH2 activity in NIH3T3 cells after OGD. * *p* < 0.05 compared with the CON group. (**D**). Western blot analysis of ALDH2 in NIH3T3 cells. (**E**) Quantification of (**D**). * *p* < 0.05, ** *p* < 0.01 compared with the control group, ^#^ *p* < 0.05 compared with the OGD group. (**F**) Changes in ALDH2 activity in NIH3T3 cells with the agonist/inhibitor treatment. * *p* < 0.05, ** *p* < 0.01 compared with the CON group, ^#^ *p* < 0.05 compared with the OGD group. (**G**) β-GAL activity analysis in NIH3T3 cells with the agonist/inhibitor treatment. *** *p* < 0.005 compared with the CON group, ^###^ *p* < 0.001 compared with the OGD group. (**H**) β-GAL staining in NIH3T3 cells with the agonist/inhibitor treatment. Scale bar = 50 μm. (**I**) Western blot analysis of p53, p21, and p16 in NIH3T3 cells. (**J**) Quantification of (**I**). ** *p* < 0.01, *** *p* < 0.001 compared with the CON group, ^#^ *p* < 0.05 compared with the OGD group. (**K**) RT-qPCR analysis of IL6, HMGB1 and TNFα mRNA in NIH3T3 cells. * *p* < 0.05, ** *p* < 0.01, *** *p* < 0.001 compared with the CON group, ^#^ *p* < 0.05, ^##^ *p* < 0.01 compared with the OGD group. (**L**) Cell proliferation was detected using ki67 staining. The red fluorescence in the image indicates the Ki67 positive cells, while the blue fluorescence indicates the nucleus. Scale bar = 50 μm. C + C: CON + CVT-10216, C + A: CON + Alda-1, O + C: OGD + CVT-10216, O + A: OGD + Alda-1.

The expression of ALDH2 protein in fibroblasts was examined through Western blot analysis after 4 h of OGD. The results, displayed in Figure 4A,B, reveal a significant up-regulation of ALDH2 protein expression in the OGD group compared to the CON group. As ALDH2 functions primarily through its enzymatic activity, we further investigated the enzyme activity of ALDH2. Figure 4C demonstrates a significant down-regulation of ALDH2 enzyme activity in the OGD group compared to the CON group. To determine the impact of ALDH2 protein expression and enzyme activity on fibroblast senescence induced through OGD, we tested the effectiveness of the ALDH2-specific agonist Alda-1 and the specific inhibitor CVT-10216. Western blot results (Figure 4D,E) showed that 10 μM CVT-10216 significantly inhibited the expression level of ALDH2 protein and the activity of ALDH enzyme under CON conditions and OGD conditions. The 20 μM ALDH2-specific agonist Alda-1 could significantly increase the expression level of ALDH2 protein under OGD conditions. The results of CCK8 showed that the two drugs had no significant effect on cell viability at this concentration (Figure S2). Based on the analysis of ALDH2 enzyme activity, as displayed in Figure 4F, it was observed that 10 μM CVT-10216 significantly inhibited ALDH2 enzyme activity under normal conditions, and 20 μM Alda-1 significantly increased ALDH2 enzyme activity under both CON and OGD conditions. ALDH2 activation significantly inhibited β-GAL activity and reduced the number of cells stained positive for β-GAL during OGD conditions, as indicated by the β-GAL activity test and staining results (Figure 4G,H). Moreover, the Western blot results (Figure 4I,J) demonstrated that ALDH2 activation significantly inhibited the expression of p53, p21, and p16 proteins associated with cell aging during OGD. The RT-qPCR results (Figure 4K) showed that the activation of ALDH2 significantly inhibited the expression of SASPs mRNA during OGD conditions. The results of Ki67 staining (Figure 4L) showed that the activation of ALDH2 could significantly increase the number of Ki67 positive cells after OGD. These results indicate that ALDH2 enzyme activity is a crucial factor in regulating fibroblast senescence.

### 3.5. Activation of ALDH2 Improves Mitochondrial Damage Induced by OGD

**Figure 5:**
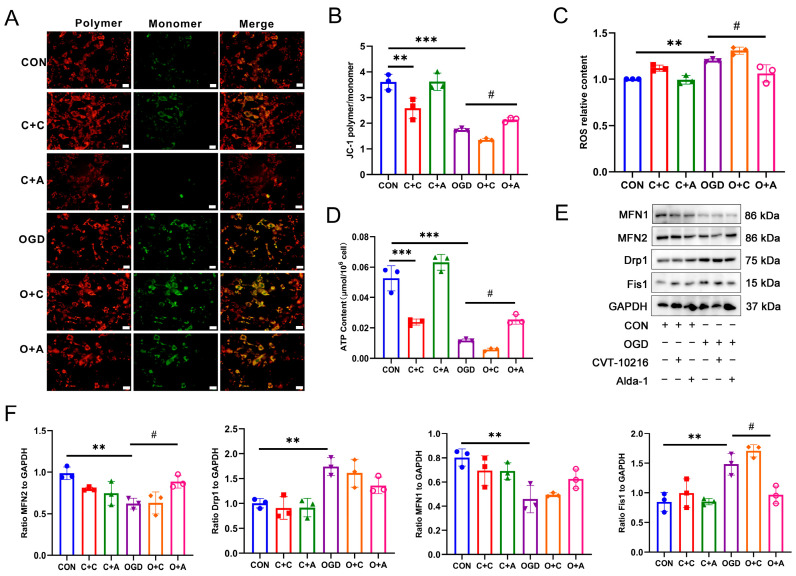
Effect of ALDH2 activity on mitochondrial damage induced by OGD. (**A**) Mitochondrial membrane potential was detected using JC-1 staining. The red fluorescence indicates the JC-1 polymer and the green fluorescence indicates the JC-1 monomer. Scar bar = 50 μm. (**B**) Quantification of (**A**). ** *p* < 0.01, *** *p* < 0.001 compared with the CON group, ^#^ *p* < 0.05 compared with the OGD group. (**C**) ROS relative content in NIH3T3 cells. ** *p* < 0.01 compared with the CON group, ^#^ *p* < 0.05 compared with the OGD group. (**D**) ATP content in NIH3T3 cells. *** *p* < 0.001 compared with the CON group, ^#^ *p* < 0.05 compared with the OGD group. (**E**) Western blot analysis of MFN1, MFN2, Drp1, and Fis1 in NIH3T3 cells. (**F**) Quantification of (**E**). ** *p* < 0.01 compared with the CON group, ^#^ *p* < 0.05 compared with the OGD group. C + C: CON + CVT-10216, C + A: CON + Alda-1, O + C: OGD + CVT-10216, O + A: OGD + Alda-1.

Mitochondrial dysfunction is a crucial factor that leads to cell senescence. ALDH2, an enzyme present in mitochondria, plays a key role in mitochondrial health. With this in mind, we conducted a study to investigate the effect of ALDH2 on the mitochondria of fibroblasts treated with OGD. JC-1 is a fluorescent dye that can be used to observe the health of mitochondria. When the mitochondria are healthy, JC-1 accumulates in the mitochondrial matrix and forms polymers that produce red fluorescence. However, if the mitochondria are damaged, the mitochondrial membrane potential will decrease significantly, and JC-1 will not be able to accumulate in the matrix. In such cases, JC-1 exists as a monomer and produces green fluorescence instead. The JC-1 staining results showed (Figure 5A,B) that the mitochondrial membrane potential of the cells in the CON + CVT-10216 group was significantly lower than that of the CON group. After OGD treatment, the mitochondrial membrane potential decreased significantly, but increased significantly in the OGD + Alda-1 group. As shown in Figure 5C, the detection results of mitochondrial ROS content indicate that the ROS level in the CON + CVT-10216 and OGD groups was significantly increased, while the ROS level in the OGD + Alda-1 group was significantly decreased. Furthermore, the intracellular ATP detection results presented in Figure 5D demonstrate that, compared to the CON group, the ATP content in CON + CVT-10216 and OGD groups was significantly reduced, while the ATP content in the OGD + Alda-1 group was considerably increased. These findings suggest that a decrease in ALDH2 activity can lead to mitochondrial damage, whereas the activation of ALDH2 can help reduce the mitochondrial damage caused by OGD and increase mitochondrial energy production. Mitochondria can remove damaged and dysfunctional mitochondria through fission and fusion. We conducted a study to explore whether ALDH2 plays a role in regulating mitochondrial fusion and fission. According to the results of the Western blot, as displayed in Figure 5E,F, the treatment with OGD resulted in decreased levels of mitochondrial fusion proteins MFN1 and MFN2, while increasing the levels of mitochondrial fission proteins Drp1 and Fis1, when compared to the CON group. Conversely, the activation of ALDH2 resulted in the increased expression of MFN2 and decreased expression of Fis1, but did not significantly impact the expression of MFN1 and Drp1. These findings suggest that ALDH2 activation may be able to regulate mitochondrial fusion and fission to some degree.

### 3.6. OGD-Induced HSPA8 Translocation to Mitochondria to Inhibit ALDH2 Enzyme Activity

**Figure 6:**
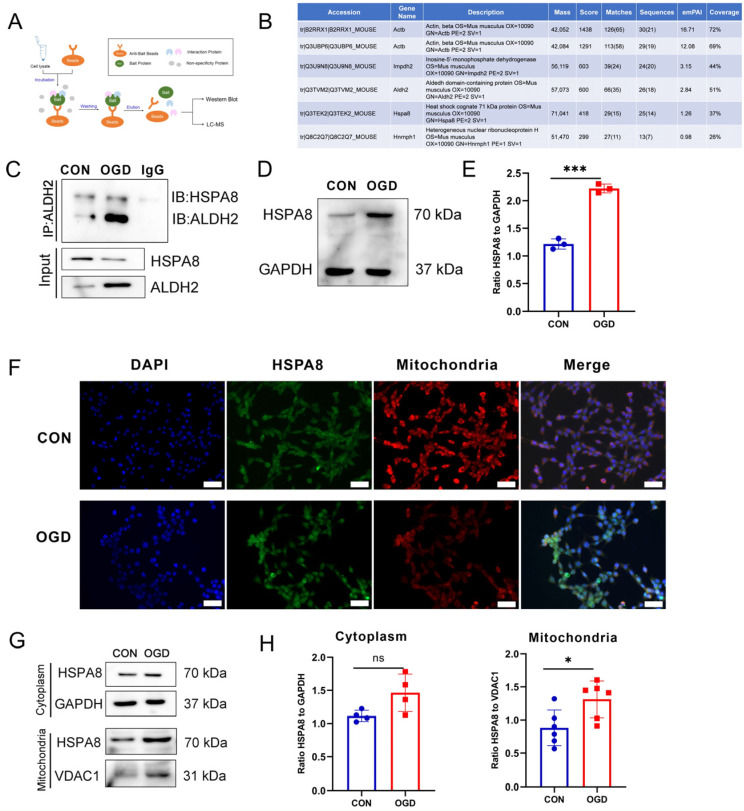
HSPA8 translocation to mitochondria to inhibit ALDH2 enzyme activity after OGD. (**A**) Co-IP-MS detection principle and workflow. (**B**) The top five proteins binding to ALDH2 under OGD conditions. (**C**) Subcellular fractionation of NIH3T3 cells treated with OGD for 4 h were immunoprecipitated using ALDH2 antibody, and bound proteins were analyzed using Western blot. (**D**) Western blot analysis of HSPA8 in NIH3T3 cells. (**E**) Quantification of (**D**). *** *p* < 0.001 compared with the Control (CON) group. (**F**) Immunofluorescence of HSPA8 in NIH3T3 cells. The green fluorescence indicates HSPA8 protein and the red fluorescence indicates mitochondria. Scale bar = 50μm. VDAC1: voltage-dependent anion channel 1, mitochondrial marker protein. (**G**) Cell mitochondria were isolated and HSPA8 protein expression was detected using Western blot. (**H**) Quantification of (**G**). * *p* < 0.05 compared with the CON group.

To identify the key factors that inhibit ALDH2 enzyme activity after OGD, Co-IP-MS was used to detect proteins that interact with ALDH2. The mass spectrometry results showed (Figure 6A,B) that Actβ, Impdh2, HSPA8, and Hnmph1 were obviously bound to ALDH2. Previous studies have found that HSPA8 was significantly increased in ischemic myocardial tissue. As a result, we chose to investigate the impact of HSPA8 on ALDH2 enzyme activity [41,42]. A Co-IP test was conducted to verify the mass spectrum detection results, and the results showed (Figure 6C) that the combination of ALDH2 and HSPA8 was enhanced after OGD compared with the CON group. The results in Figure 6D,E demonstrate a significant increase in HSPA8 protein expression after OGD compared to the CON group. The immunofluorescence results (Figure 6F) further corroborated this finding by indicating a significant enhancement in the green fluorescence of the HSPA8 protein. In order to investigate the correlation between HSPA8 elevation subsequent to OGD and its movement to the mitochondria where it can interact with ALDH2, a study was conducted to separate cell mitochondrial proteins and utilize a Western blot to identify HSPA8 expression within the mitochondria. The obtained results (Figure 6G,H) demonstrated a considerable rise in HSPA8 inside the mitochondria following OGD. 

### 3.7. Inhibition of HSPA8 Can Improve Fibroblast Senescence by Increasing ALDH2 Activity after OGD

**Figure 7:**
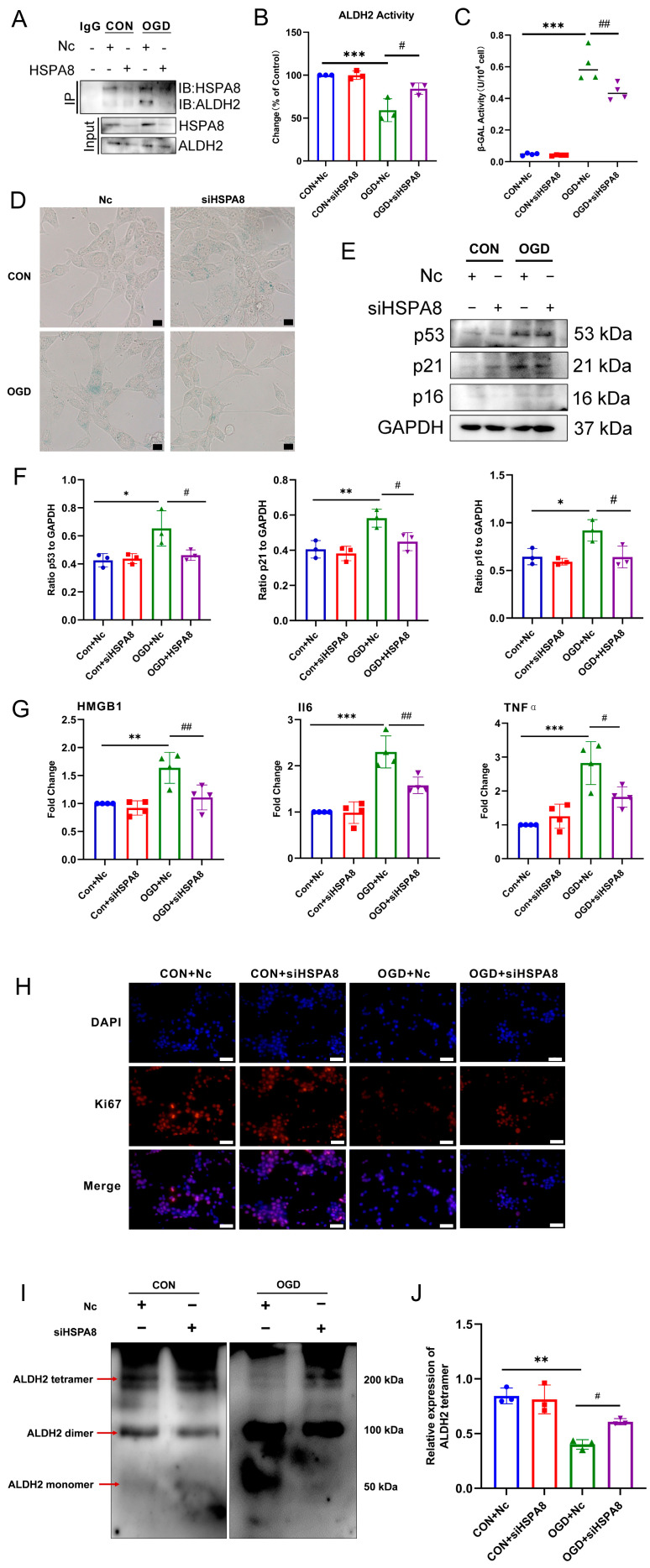
Inhibition of HSPA8 alleviates OGD-induced fibroblasts senescence. (**A**) Subcellular fractionation of NIH3T3 cells treated with OGD for 4 h were immunoprecipitated using HSPA8 antibody, and bound proteins were analyzed using Western blot. (**B**) Changes in ALDH2 activity in NIH3T3 cells with the siHSPA8 transfection. *** *p* < 0.001 compared with the Con + Nc group. ^#^ *p* < 0.05, compared with the OGD + Nc group. (**C**) β-GAL activity analysis in NIH3T3 cells with the siHSPA8 transfection. *** *p* < 0.001compared with the Con + Nc group, ^##^ *p* < 0.01 compared with the OGD + Nc group. (**D**) β-GAL staining in NIH3T3 cells with the siHSPA8 transfection. (**E**) Western blot analysis of p53, p21, and p16 in NIH3T3 cells. (**F**) Quantification of (**E**). * *p* < 0.05, ** *p* < 0.01 compared with the Con + Nc group. ^#^ *p* < 0.05 compared with the OGD + Nc group. (**G**) RT-qPCR analysis of IL6, HMGB1, and TNF-α mRNA in NIH3T3 cells. ** *p* < 0.01, *** *p* < 0.001 compared with the Con + Nc group. ^#^ *p* < 0.05, ^##^ *p* < 0.01 compared with the OGD + Nc group. (**H**) Cell proliferation was detected using Ki67 staining. The red fluorescence in the image indicates the Ki67 positive cells, while the blue fluorescence indicates the nucleus. Scale bar = 50 μm. (**I**) Cell lysates were treated with disuccinimidyl suberate (DSS) for 30 min and protein levels of ALDH2 were examined. (**J**) Quantification of (**I**). ** *p* < 0.01 compared with the CON group, ^#^ *p* < 0.05 compared with the OGD group. Nc: negative control.

The Co-IP results (Figure 7A) showed that after inhibiting the expression of HSPA8, the binding of HSPA8 and ALDH2 in the OGD group was significantly reduced. The expression of HSPA8 in NIH3T3 cells was inhibited by siRNA transfection, and 48 h was first determined as the optimal transfection time (Figure S3). Based on the results of Figure 7B, it was found that inhibiting the expression of HSPA8 resulted in a significant increase in the activity of the ALDH2 enzyme in the OGD group. This suggests that reducing the binding of HSPA8 to ALDH2 after OGD by inhibiting its expression in cells can improve the enzyme activity of ALDH2. Subsequently, we determined whether inhibiting HSPA8 to increase ALDH2 enzyme activity would improve the premature senescence of fibroblasts caused by OGD. The results of β-GAL enzyme activity detection (Figure 7C) showed that the inhibition of HSPA8 could significantly inhibit the β-GAL enzyme activity after OGD, and the results of β-GAL staining (Figure 7D) also confirmed this. The detection results of cell senescence marker protein in the Western blot analysis showed the following (Figure 7E,F): Compared with the OGD + Nc group, the expression of p53, p21, and p16 in the OGD + siHSPA8 group was significantly down-regulated. Additionally, the RT-qPCR results (Figure 7G) demonstrated that inhibiting HSPA8 significantly lowered the expression of the SASPs factors, IL6, HMGB1, and TNF-α mRNA after OGD. The results of Ki67 staining (Figure 7H) showed that the inhibition of HSPA8 could significantly increase the number of Ki67 positive cells after OGD. These results suggest that inhibiting the expression of HSPA8 and reducing the binding of HSPA8 to mitochondrial ALDH2 under OGD conditions could help improve the decrease in ALDH2 enzyme activity caused by OGD. This effect can significantly inhibit the premature senescence of fibroblasts induced by OGD. Research has revealed that only the tetramer form of ALDH2 exhibits enzyme activity [26]. Therefore, it is speculated whether the expression of HSPA8 affects the tetramer formation of ALDH2 post OGD. The results of the DSS crosslinking experiment indicated that the ALDH2 dimer was significantly increased in the OGD group compared to the CON group. Additionally, inhibiting the expression of HSPA8 led to a significant increase in the ALDH2 tetramer in the OGD group. These findings suggest that OGD treatment causes an increase in the inactive ALDH2 dimer, reduces the binding of HSPA8 to ALDH2, promotes the formation of ALDH2 tetramer, and boosts its activity.

## 4. Discussion

The occurrence of MI is significantly threatened by cellular senescence, which is a key factor contributing to high mortality rates. Given that cardiovascular disease is associated with senescent cells, avoiding the risk factors that induce cellular senescence, eliminating senescent cells, and attenuating the SASPs have emerged as attractive therapeutic strategies [43].

Cardiac fibroblasts are essential in cardiac repair, scar formation, fibrosis, and pathological reconstruction after MI [44]. In recent years, it has been found that some myocardial fibroblasts will undergo cellular senescence after myocardial injury [45,46,47]. Our research used bioinformatics analysis to discover that the cell senescence signaling pathway was activated 3 days after MI. In comparison, the apoptosis signaling pathway was significantly activated 7 days after MI, suggesting that fibroblast senescence was first induced in the early stage of MI. Myocardial infarction is primarily caused by interrupted blood flow, which results in a lack of oxygen and nutrients in the myocardial tissue. To recreate this environment in vitro, OGD treatment is frequently employed [48]. Previous research conducted by Lim and colleagues demonstrated that cellular senescence can be induced using oxygen and glucose deprivation/reoxygenation (OGD/R) [49]. Furthermore, Feng et al. have shown that neonatal rat cardiomyocytes may experience hypoxia-induced senescence in vitro [50]. These findings suggest that the absence of oxygen and glucose may be critical factors contributing to cell senescence. For the current study, the cells underwent OGD treatment for various time intervals (0 h, 2 h, 4 h, and 8 h) to observe changes in cell fate. The outcomes of the experiment revealed that an OGD treatment for 4 h could significantly induce senescence in NIH3T3 cells. On the other hand, OGD treatment for 8 h caused a large number of cell apoptosis. These results suggested that the duration of the OGD treatment has different effects on cell fate. However, the molecular mechanism responsible for this phenomenon has yet to be reported. This paper aimed to explore the molecular mechanism of 4 h OGD-induced cell senescence.

The further data analysis revealed the significant role of mitochondrial aldehyde dehydrogenase ALDH2 in regulating cell senescence. Prior research has established that activating ALDH2 can effectively aid in repairing myocardial infarction injuries, specifically in the maintenance of mitochondrial homeostasis [51,52,53]. Recent studies have shown that ALDH2 plays a crucial role in reducing the risk of cardiovascular disease by regulating cellular senescence. Nannelli et al. discovered that when ALDH2 function is impaired, it could lead to premature endothelial senescence, which manifests in cell dysfunction, altered metabolism, and an increased likelihood of age-related cardiovascular disease [20] Zhu et al. found that a deficiency in ALDH2 may potentially accelerate vascular smooth muscle cell senescence by promoting plaque instability through mROS. However, the correlation between ALDH2 function and fibroblast senescence remains unclear. In our research, the expression of ALDH2 in myocardial fibroblasts and NIH3T3 cells changed significantly after MI and OGD, respectively. However, the decline in ALDH2 enzyme activity could be the primary factor in fibroblast senescence. The activation of ALDH2 demonstrated an ability to effectively prevent the senescence of NIH3T3 cells. 

Numerous studies have indicated that cellular senescence is associated with significant changes in the structure and function of mitochondria. It is generally accepted that impaired mitochondria can either merge with healthy ones to dilute and restructure the mitochondrial matrix, or undergo fission to separate from the damaged ones and be degraded. Thus, it is essential to maintain an optimal balance between the fission and fusion processes to promote optimal cellular function [54]. Mitochondrial dynamics have been linked to cell fate decisions in various cell types. The fate of cells after mitosis can be regulated by the process of mitochondrial fusion and fission [55]. These processes, combined with mitochondrial autophagy to remove damaged mitochondria, play a crucial role in controlling the occurrence and development of diseases [56]. Unbalanced mitochondrial fusion and fission may accelerate cellular senescence. Therefore, proper intervention in mitochondrial fusion and fission can delay the process of cellular senescence.

According to our research findings, administering an OGD treatment resulted in a noteworthy reduction in the mitochondrial membrane potential, an increase in the ROS levels, and a decline in the ATP production in fibroblasts, accompanied by a significant rise in the mitochondrial fission protein and a significant drop in the mitochondrial fusion protein. These changes were found to be influenced by the activity of the ALDH2 enzyme. Interestingly, a similar phenomenon was reported in senescent endothelial cells [20]. Our study uncovered a novel finding that the activation of ALDH2 could potentially have a positive effect on mitochondrial fusion and fission in fibroblasts under OGD conditions. Hence, the impairment of mitochondrial function due to ALDH2 inactivation could be a crucial factor in triggering cell senescence.

In order to identify the key factors that cause the inhibition of ALDH2 enzyme activity, we detected the intracellular proteins that interact with ALDH2 after OGD through co-immunoprecipitation and mass spectrometry, among which HSPA8 had a high abundance. Yu et al. showed that HSPA8 regulated the macrophage-mediated inflammatory response during cardiac remodeling [57]. In rat pulmonary vein cardiomyocytes, HSPA8 can be a helper protein in hyperpolarization activation of chloride ion channels and may be involved in regulating atrial fibrillation [58]. Further investigation is needed to fully understand the role of HSPA8 in repairing myocardial infarction. Previous studies have indicated that when cells are stimulated, HSPA8 may relocate to the mitochondria and regulate their function, ultimately impacting cell fate, including senescence [59,60]. In our study, it was found that after 4 h of OGD treatment, HSPA8 in fibroblasts translocated to mitochondria and bound to ALDH2, which resulted in a decrease in the activity of the ALDH2 enzyme. By inhibiting the expression of HSPA8 and reducing its binding with ALDH2, the enzyme activity of ALDH2 could be significantly improved, thus inhibiting cell senescence. Previous studies have shown that the activity of the ALDH2 enzyme can be affected by its tetramer/dimer formation [61]. We thoroughly investigated whether HSPA8 affects the structure of ALDH2 after OGD. Our research revealed that OGD resulted in an increase in inactive ALDH2 dimers. This could explain the increase in ALDH2 expression and the decrease in enzyme activity after OGD. However, inhibiting HSPA8 can increase the tetramer of ALDH2 after OGD, thereby increasing its enzyme activity.

In summary, our findings suggested that blocking HSPA8 could improve mitochondrial ALDH2 enzyme activity, relieve the fibroblast senescence caused by OGD, and decrease the levels of the p53, p21, and p16 proteins, SASPs mRNA expression, and β-galactosidase activity, while increasing the number of Ki67-positive cells. This new understanding of cellular senescence mechanisms is a significant step towards improving myocardial infarction injury. Further in vivo studies are needed to confirm the results.

## Figures and Tables

**Table 1:** Primer sequences for RT-qPCR.

Gene Symbol	Accession	Species	Forward	Reverse
TNFα	NM_001278601.1	Mouse	GGACTAGCCAGGAGGGAGAACAG	GCCAGTGAGTGAAAGGGACAGAAC
IL6	NM_001314054.1	Mouse	CTTCTTGGGACTGATGCTGGTGAC	TCTGTTGGGAGTGGTATCCTCTGTG
HMGB1	NM_010439.4	Mouse	AGGCTGACAAGGCTCGTTATGAAAG	GGGCGGTACTCAGAACAGAACAAG
GAPDH	NM_001411841.1	Mouse	GGCAAATTCAACGGCACAGTCAAG	TCGCTCCTGGAAGATGGTGATGG

## Data Availability

Data are contained within the article and Supplementary Materials.

## Supplementary Materials

The following supporting information can be downloaded at: https://www.mdpi.com/article/10.3390/antiox13010042/s1, Figure S1: Expression of ALDH2, PKM and PFKL genes in myocardial cells of mice; Figure S2: Cytotoxicity in NIH3T3 cells after 24h treatment of Alda-1 and CVT-10216; Figure S3: Inhibitory effect of transfection with small interfering RNA (siRNA) on HSPA8.

## Abbreviations

MI, myocardial infarction; OGD, oxygen-glucose deprivation; ALDH2, aldehyde dehydrogenase 2; MFN1, Mitofusin-1; MFN2, Mitofusin-2; Drp1 Dynamin-related protein 1; Fis1, fission, Mitochondrial 1; HSPA8, Heat Shock 70-kDa Protein 8; Co-IP, co-immunoprecipitation; MS, mass spectrometry; DSS, disuccinimidyl suberate.
