# Supplementary material for: The Binding of HSPA8 and Mitochondrial ALDH2 Mediates Oxygen-Glucose Deprivation-Induced Fibroblast Senescence
Source: Antioxidants (Basel). 2023 Dec 25;13(1):42. doi: 10.3390/antiox13010042 (PMC10812545; doi:10.3390/antiox13010042)
Supplement: Supplementary file 1 [file antioxidants-13-00042-s001.zip › antioxidants-2717961-supplementary.pdf]

Supplementary Figures

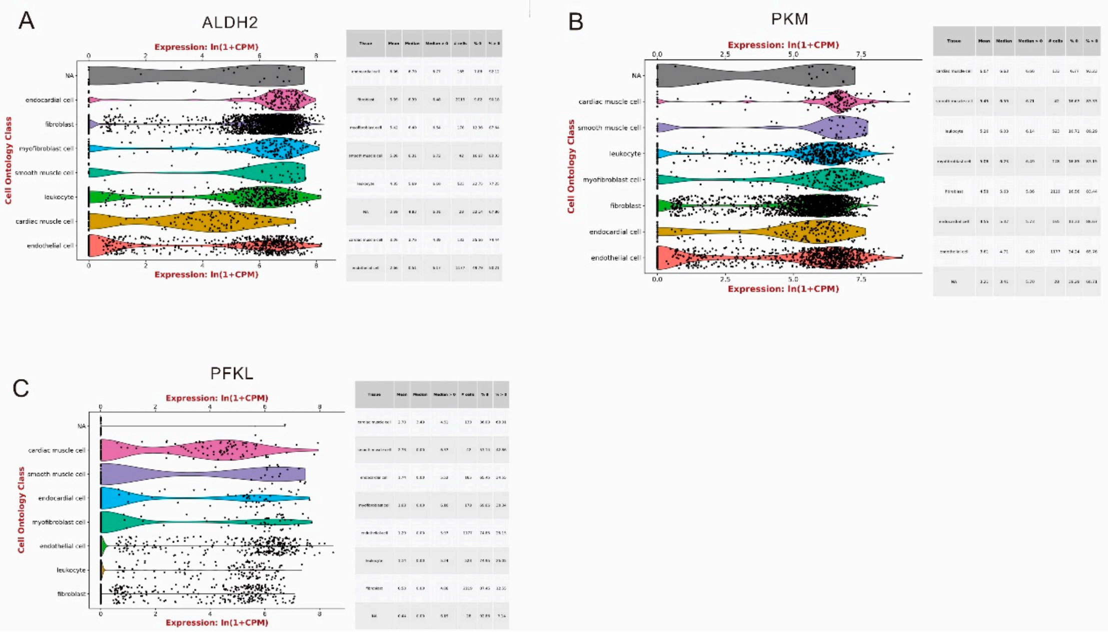

Supplementary Figure S1. Expression of ALDH2, PKM and PFKL genes in myocardial cells of mice. (A) Expression of ALDH2 in myocardial tissue of mice. (B) Expression of PKM in myocardial tissue of mice. (C) Expression of PFKL in myocardial tissue of mice.

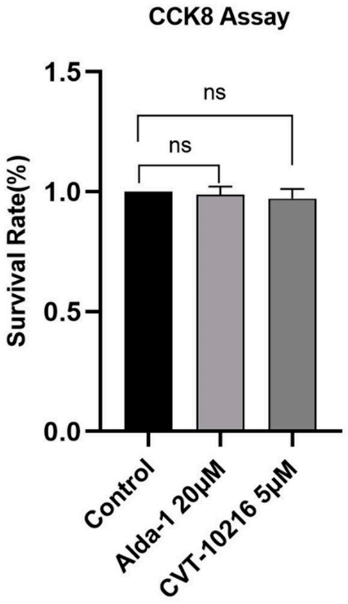

Supplementary Figure S2. Cytotoxicity in NIH3T3 cells after 24h treatment of Alda-1 and CVT-10216. The effect of ALDH2 agonist Alda-1 and inhibitor CVT-10216 on NIH3T3 cell survival rate was determined by CCK8 assay.

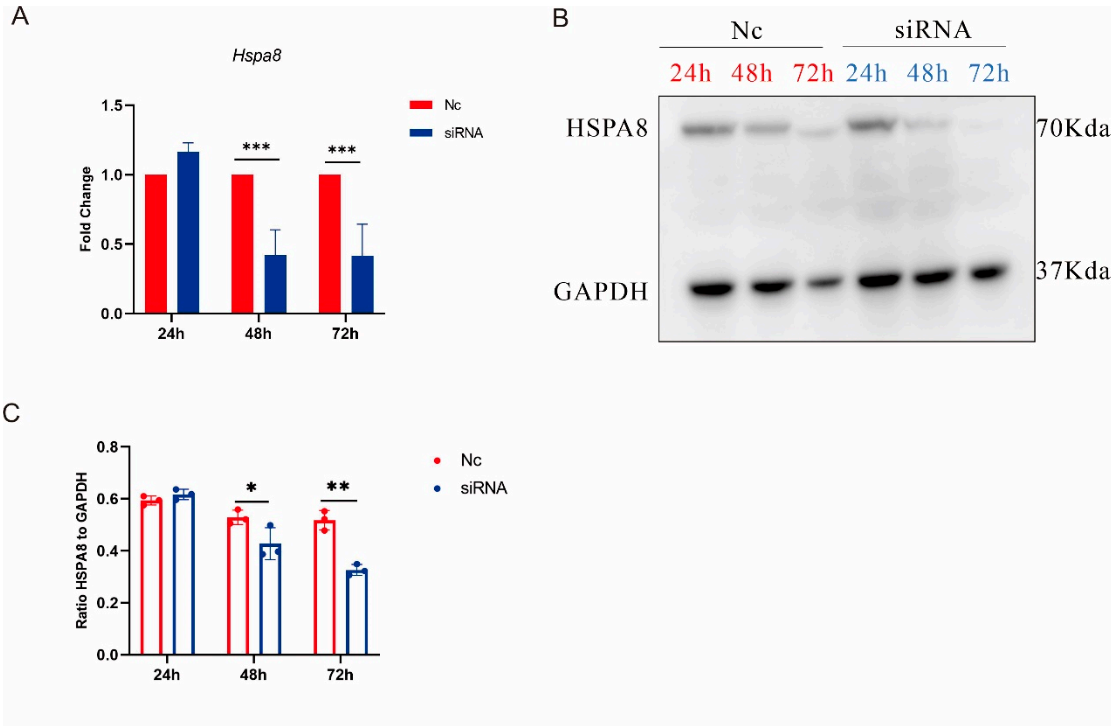

Supplementary Figure S3. Inhibitory effect of transfection with small interfering RNA (siRNA) on HSPA8. (A) The effect of transfection time on HSPA8 mRNA expression was detected by RT-qPCR. (B) The effect of transfection time on HSPA8 protein expression was detected by Western Blot. (C) Quantification of (B). *$p < 0.05$, **$p < 0.01$, *** $p < 0.001$ compared with the Nc group.
